# L-cysteine improves blood fluidity impaired by acetaldehyde: *In vitro* evaluation

**DOI:** 10.1371/journal.pone.0214585

**Published:** 2019-03-29

**Authors:** Ippo Otoyama, Hironobu Hamada, Tatsushi Kimura, Haruchi Namba, Kiyokazu Sekikawa, Norimichi Kamikawa, Teruki Kajiwara, Fumiya Aizawa, Yoshinobu M Sato

**Affiliations:** 1 Department of Physical Analysis and Therapeutic Sciences, Graduate School of Biomedical and Health Sciences, Hiroshima University, Hiroshima, Japan; 2 Faculty of Early Childhood Education and Care, Ohkagakuen University, Aichi, Japan; Universidade de Lisboa Instituto Superior Tecnico, PORTUGAL

## Abstract

Blood fluidity is reportedly influenced by the volume and function of blood cells and plasma and is a predictor of primary cardiovascular events in patients with traditional cardiovascular risk factors. Heavy alcohol consumption was shown to be associated with a higher risk for cardiovascular diseases. Acetaldehyde (ACD), an oxidizing substance formed from ethanol, reportedly stimulates monocyte adhesion, causes abnormalities in the red blood cell (RBC) membrane, and decreases RBC deformability. In addition, it was reported that blood ACD levels are reduced in mice pretreated with L-cysteine. However, there are no studies on the effect of ACD and/or L-cysteine on blood fluidity. In the present study, we evaluated whether ACD impairs blood fluidity. In addition, the effect of L-cysteine on blood fluidity impaired by ACD was examined. Blood samples were obtained from 10 healthy, non-smoking, male volunteers (age: 23.4 ± 1.2 years, body mass index: 21.8 ± 2.6 kg/m^2^). ACD or ACD and L-cysteine were added to the blood samples before each experiment. We measured the passage time of 100 μL blood and RBC suspension using Kikuchi’s microchannel method. Percentage of microchannel obstruction and the number of adherent white blood cells (WBCs) on microchannel terrace were counted. The blood passage time, percentage of microchannel obstruction, and numbers of adherent WBCs on the microchannel terrace increased after adding ACD in a concentration-dependent manner, whereas they decreased after adding ACD and L-cysteine in a L-cysteine concentration-dependent manner. No significant effects were observed in passage time for 100 μL RBC suspension after adding ACD and L-cysteine. This study suggested that blood fluidity impaired by ACD might improve after adding L-cysteine.

## Introduction

Blood fluidity, as a reciprocal of viscosity, is reported to be influenced by the volume and function of red blood cells (RBCs), white blood cells (WBCs), platelets, and plasma [1–4]. Micro-channel array flow analyzer (MC-FAN, Hitachi Haramachi Electronics Co. Ltd., Japan) is generally accepted as a reliable tool for assessing whole blood fluidity [5–7]. Blood passage time, measured using MC-FAN, is one of the parameters of blood fluidity. It was reported that blood passage time is a predictor of primary cardiovascular events in patients with traditional cardiovascular risk factors [8].

Heavy alcohol consumption, which leads to increased blood viscosity, decreased RBC deformability, and impaired fibrinolytic potential [9,10], was reported to be associated with a higher risk for cardiovascular diseases and ischemic cerebrovascular diseases [11,12]. Especially, acetaldehyde (ACD), which is an oxidizing substance formed from ethanol, stimulates monocyte adhesion [13], causes abnormalities in the RBC membrane [14], and decreases RBC deformability in patients with liver cirrhosis [15]. However, to the best of our knowledge, there are no studies on the effects of ACD on blood fluidity.

L-cysteine, which is an amino acid, has effects on attenuating inflammatory responses and restoring the susceptibility of activated immune cells to apoptosis [16]. A previous study reported that pre-administration of L-cysteine decreased serum ACD levels in mice, which were given ethanol [17].

In the present study, we hypothesized that ACD impairs blood fluidity and that L-cysteine improves ACD-induced blood fluidity impairment. The purpose of this study was to clarify whether ACD increases blood passage time. In addition, the effect of L-cysteine on blood passage time increased by ACD was examined.

## Materials and methods

### Subjects

We recruited healthy, non-smoking, male volunteers. Subjects who have abnormal blood characteristics and diseases such as cardiac diseases, diabetes mellitus, hypertension, and infectious diseases were excluded. Written informed consent was obtained from all the subjects before the investigation was conducted. This study was approved by the Ethical Committee for Epidemiology of Hiroshima University (Approval No. E-487). The study was conducted according to the principles expressed in the Declaration of Helsinki.

### Blood collection

Blood samples were collected from an antecubital vein in tubes containing heparin solution (5% v/v 1000 IU/mL heparin). The subjects were prohibited from eating and drinking for two hours before blood collection. In addition, alcohol was prohibited starting from the night before the trial day.

### Preparation of RBC suspension

The blood was added to the Lymphocyte Separation Solution (d = 1.119, Nacalai Tesque, Inc., Kyoto, Japan) and centrifuged at 400 × *g* for 30 min. After centrifugation, plasma, buffy coat, and Lymphocyte Separation Solution were removed to obtain RBCs. RBCs were washed with phosphate buffered saline (PBS, Wako Pure Chemical Industries, Ltd., Osaka, Japan) containing 0.1% albumin. We prepared an RBC suspension with 50% hematocrit level using PBS containing 0.1% albumin.

### Blood sample preparation

ACD (Nacalai Tesque, Inc., Kyoto, Japan) and L-cysteine (FUJIFILM Wako Pure Chemical Corporation, Osaka, Japan) were dissolved in normal saline. Ten microliters of normal saline with ACD or L-cysteine was added to 1000 μL of blood samples, whereas 10 μL of normal saline was added to control samples. L-cysteine was added to blood samples and mixed by inverting the tube for 10 s. Then, ACD was immediately added and mixed again. Blood passage time was measured in 5 min after adding ACD. The final concentration of ACD was 1, 3, and 5 mM and that of L-cysteine was 0.5, 1, and 2.5 mM based on our preliminary study (data not shown). All experiments were performed under 19°C because the boiling temperature of ACD is 20.2°C.

### Determination of blood passage time and RBC suspension passage time

The passage time for 100 μL blood and RBC suspension was assessed using MC-FAN and microgrooves (width, 7 μm; length, 30 μm; depth, 4.5 μm) as reported previously [4–7]. The passage time for 100 μL blood and RBC suspension was measured twice, and the average value was used for analysis.

### Determination of microchannel obstruction and WBC adhesion

Microscopic view of blood flow was stored on a digital recorder for off-line analysis. Then, the percentage of microchannel obstruction and the number of adherent WBCs on microchannel terrace were counted. An investigator who was blinded to the subject’s backgrounds selected 5 images at a final volume of 20 μL blood and analyzed them.

### Statistics

All data shows mean ± standard deviation (SD). We used iterative measurement variance analysis. When a significant difference was observed, we conducted multiple comparisons by Dunnett method. We used SPSS Statistics version 23.0 for Windows (IBM Japan Ltd, Tokyo, Japan), and the differences associated with p < 0.05 were considered statistically significant.

## Results

### Subjects’ characteristics

A total of 10 healthy, non-smoking, male subjects were enrolled. The mean age and body mass index were 23.4 ± 1.2 years and 21.8 ± 2.6 kg/m^2^, respectively. The mean WBCs, RBCs, hemoglobin levels, hematocrit levels, and platelet counts were 5.5 ± 1.6 × 10^3^ cell/μL, 499.1 ± 28.3 × 10^4^ cell/μL, 15.3 ± 0.9 g/dL, 45.0 ± 2.6%, and 21.1 ± 4.6 × 10^4^ cell/μL, respectively.

### Effects of ACD and L-cysteine on blood passage time

**Fig 1:**
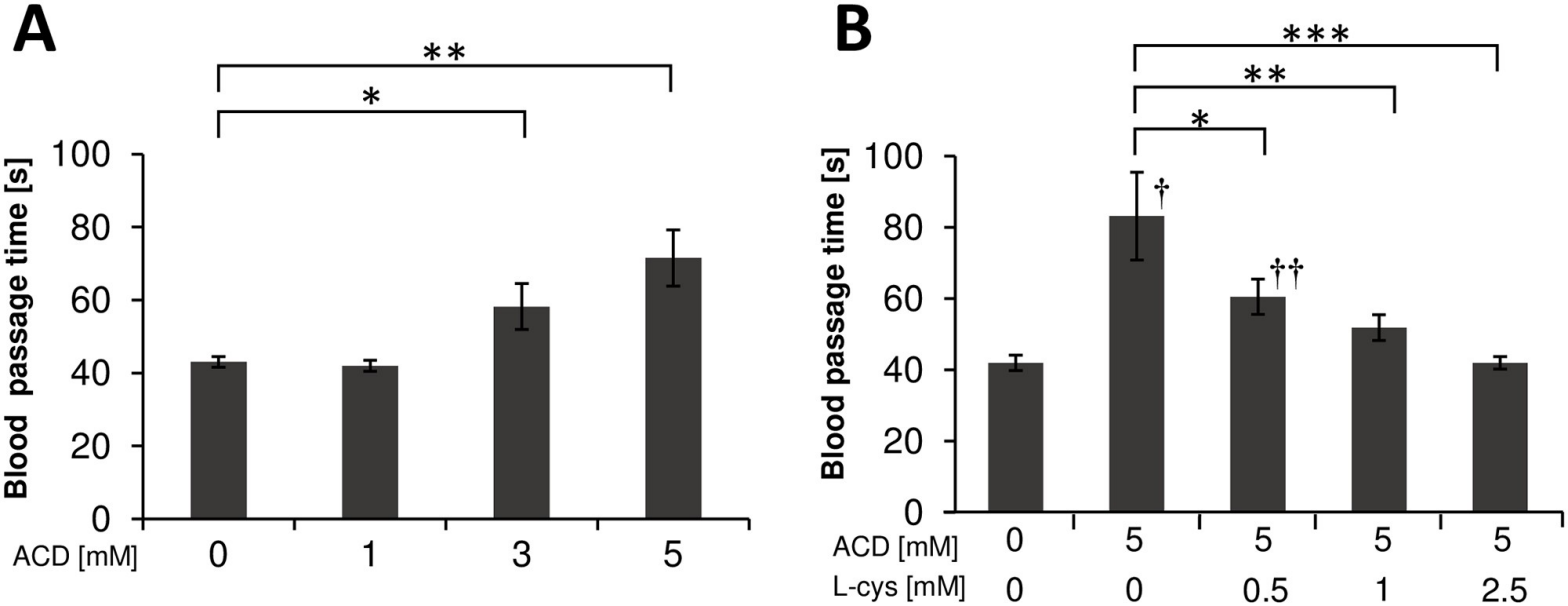
Passage time for 100 μL blood. (A) Passage time for 100 μL blood following ACD addition. *, p < 0.01, 0 mM ACD vs. 3 mM ACD; **, p < 0.001, 0 mM ACD vs. 5 mM ACD. (B) Passage time for 100 μL blood following the addition of ACD and/or L-cysteine. *, p < 0.05, 5 mM ACD with 0 mM L-cysteine vs. 5 mM ACD with 0.5 mM L-cysteine; **, p < 0.005, 5 mM ACD with 0 mM L-cysteine vs. 5 mM ACD with 1 mM L-cysteine. ***, p < 0.001, 5 mM ACD with 0 mM L-cysteine vs. 5 mM ACD with 2.5 mM L-cysteine; †, p < 0.001, 0 mM ACD with 0 mM L-cysteine vs. 5 mM ACD with 0 mM L-cysteine; ††, p < 0.05, 0 mM ACD with 0 mM L-cysteine vs. 5 mM ACD with 0.5 mM L-cysteine.

Blood passage time for 100 μL blood increased after adding ACD in a concentration-dependent manner. The blood passage time in the 3 mM ACD and 5 mM ACD treatments was significantly higher than that in the 0 mM ACD treatment (p < 0.01 and p < 0.001, respectively; Fig 1A). Blood passage time for 100 μL blood after ACD and L-cysteine addition was decreased in an L-cysteine concentration-dependent manner. The blood passage time in the 0.5, 1, and 2.5 mM L-cysteine treatments was significantly lower than that in the 0 mM L-cysteine treatment (p < 0.05, p < 0.005 and p < 0.001, respectively; Fig 1B). Blood passage time for 100 μL blood in the 5 mM ACD and 2.5 mM L-cysteine treatments was similar to that in the 0 mM ACD and 0 mM L-cysteine treatments.

### Effects of ACD and L-cysteine on microchannel obstruction and WBC adhesion

**Fig 2:**
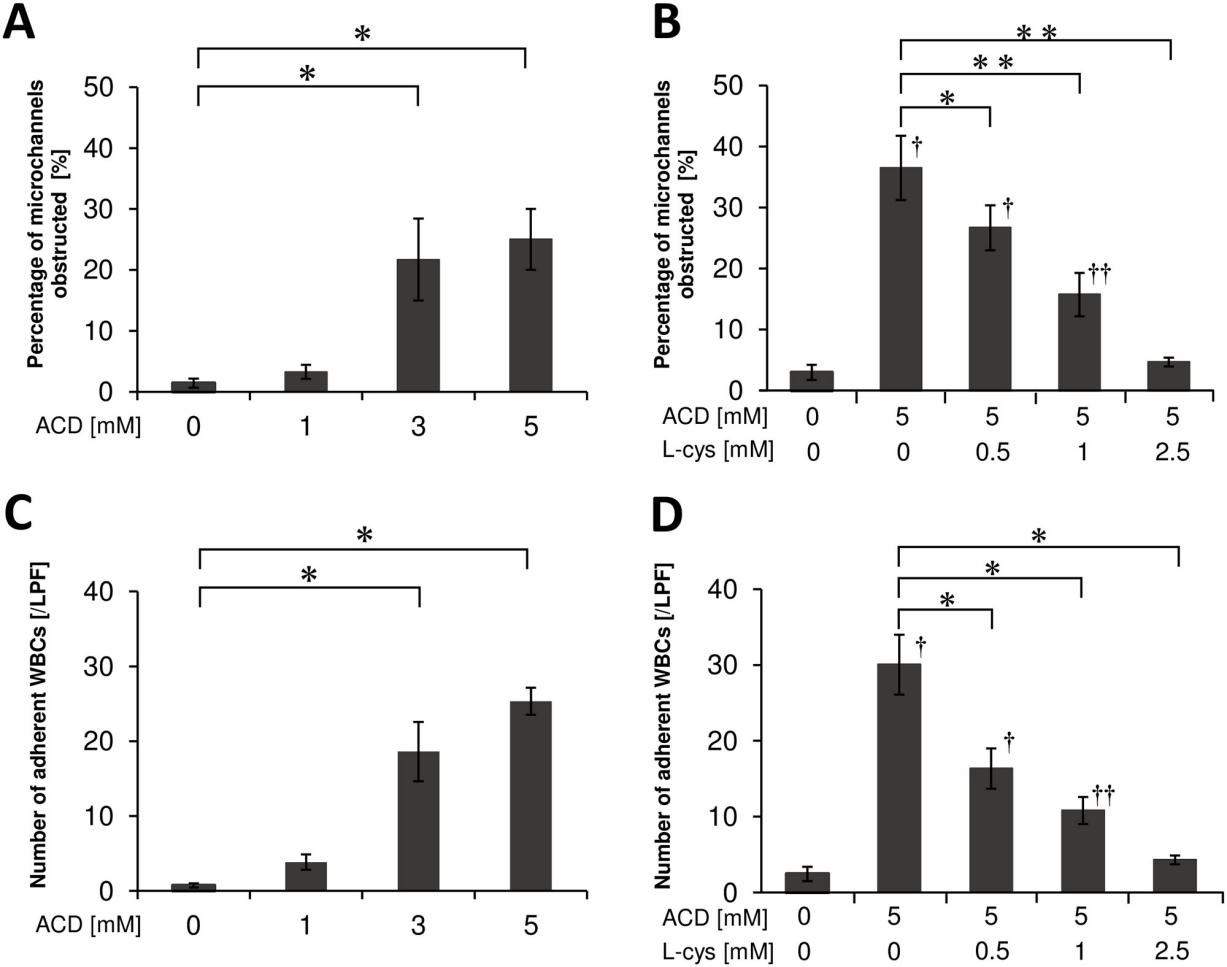
Microchannel obstruction and WBC adhesion. (A) Percentage of microchannels obstructed following ACD addition. *, p < 0.001, 0 mM ACD vs. 3 mM ACD and 5 mM ACD. (B) Percentage of microchannels obstructed following addition of ACD and/or L-cysteine. *, p < 0.05, 5 mM ACD with 0 mM L-cysteine vs. 5 mM ACD with 0.5 mM L-cysteine; **, p < 0.001, 5 mM ACD with 0 mM L-cysteine vs. 5 mM ACD with 1 mM L-cysteine and 5 mM ACD with 2.5 mM L-cysteine; †, p < 0.001, 0 mM ACD with 0 mM L-cysteine vs. 5 mM ACD with 0 mM L-cysteine and 5 mM ACD with 0.5 mM L-cysteine; ††, p < 0.005, 0 mM ACD with 0 mM L-cysteine vs. 5 mM ACD with 1 mM L-cysteine. (C) The number of adherent WBCs on the microchannel terrace following ACD addition. *, p < 0.001, 0 mM ACD vs. 3 mM ACD and 5 mM ACD. (D) The number of adherent WBCs on the microchannel terrace following addition of ACD and/or L-cysteine. *, p < 0.001, 5 mM ACD with 0 mM L-cysteine vs. 5 mM ACD with 0.5 mM L-cysteine, 5 mM ACD with 1 mM L-cysteine and 5 mM ACD with 2.5 mM L-cysteine; †, p < 0.001, 0 mM ACD with 0 mM L-cysteine vs. 5 mM ACD with 0 mM L-cysteine and 5 mM ACD with 0.5 mM L-cysteine; ††, p < 0.01, 0 mM ACD with 0 mM L-cysteine vs. 5 mM ACD with 1 mM L-cysteine. LPF = low power field.

The percentage of microchannel obstruction and numbers of adherent WBCs on the microchannel terrace increased after adding ACD in a concentration-dependent manner (Fig 2A and 2C). The percentage of microchannel obstruction in the 3 mM ACD and 5 mM ACD treatments was significantly higher than that in the 0 mM ACD treatment (all, p < 0.001). In addition, the numbers of adherent WBCs on the microchannel terrace in the 3 mM ACD and 5 mM ACD treatments were significantly increased compared to that in the 0 mM ACD treatment (all, p < 0.001).

The percentage of microchannel obstruction and numbers of adherent WBCs on the microchannel terrace after ACD and L-cysteine addition were decreased in an L-cysteine concentration-dependent manner (Fig 2B and 2D). The percentage of microchannel obstruction in the 5 mM ACD with 0.5, 1, and 2.5 mM L-cysteine treatment was significantly lower than that in the 5 mM ACD and 0 mM L-cysteine treatments (p < 0.05, p < 0.001 and p < 0.001, respectively). The numbers of adherent WBCs on the microchannel terrace in the 5 mM ACD with 0.5, 1, and 2.5 mM L-cysteine treatment was also significantly decreased compared to that in the 5 mM ACD and 0 mM L-cysteine treatments (all, p<0.001). There was no significant difference in the percentage of microchannel obstruction and numbers of adherent WBCs between 5 mM ACD and 2.5 mM L-cysteine treatments and 0 mM ACD and 0 mM L-cysteine treatments.

**Fig 3:**
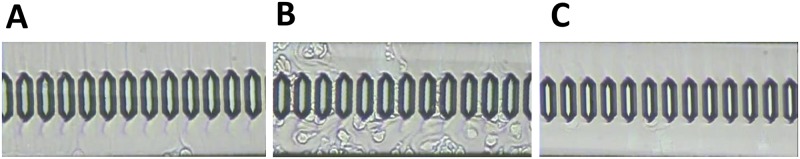
Flow path at the time of measurement. Flow path at the time of measurement with MC-FAN. (A) Flow channel images of 0 mM ACD with 0 mM L-cysteine, (B) 5 mM ACD with 0 mM L-cysteine, and (C) 5 mM ACD with 2.5 mM L-cysteine treatments.

Obstruction of microchannels by platelet aggregation and WBC adhesion was observed after adding ACD, but not after adding ACD and L-cysteine (Fig 3).

### Effects of ACD and L-cysteine on RBC suspension passage time

**Fig 4:**
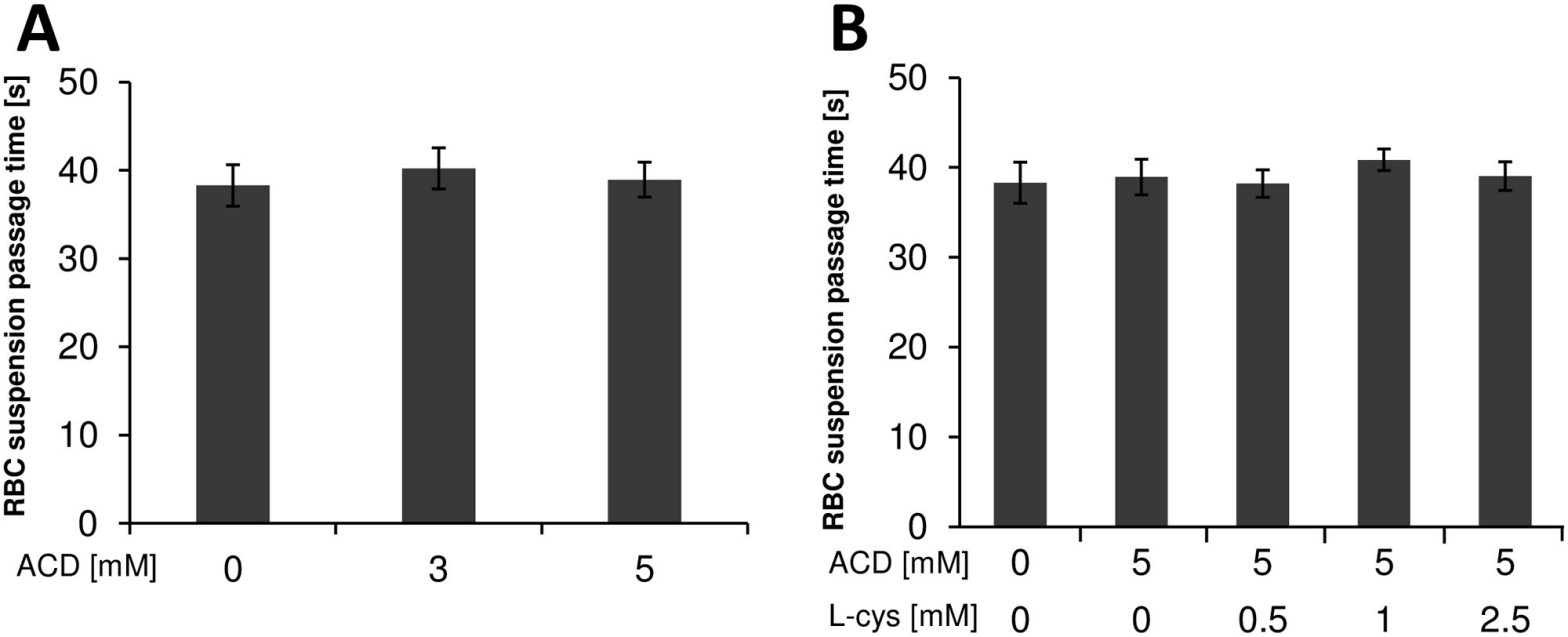
The passage time of RBC suspension. (A) RBC suspension passage time following ACD addition. (B) RBC suspension passage time following addition of ACD and/or L-cysteine.

No significant effects were observed in passage time for 100 μL RBC suspension after adding ACD and L-cysteine (Fig 4A and 4B).

## Discussion

This is an *in vitro* study to clarify the usefulness of L-cysteine in blood fluidity impaired by ACD. In the present study, the blood passage time, percentage of microchannel obstruction, and numbers of adherent WBCs on the microchannel terrace, which were increased after adding ACD, were decreased after L-cysteine addition. Blood fluidity, impaired by ACD, might improve after adding L-cysteine.

Blood fluidity may be impaired by ACD via several mechanisms such as the effect of WBC adhesion and platelet aggregation. Previous studies reported that ACD stimulated monocyte adhesion [13], caused abnormalities of membrane lipid compositions by reacting irreversibly with proteins of RBC [14], and decreased RBC deformability in patients with liver cirrhosis [15]. Based on these previous studies, we hypothesized that ACD impairs blood fluidity by decreasing RBC deformability as well as increasing WBC adhesion and platelet aggregation. To clarify the effects of ACD on blood fluidity, we measured the passage time for both RBC suspension and blood using MC-FAN. The passage time for RBC suspension obtained from healthy subjects was not increased after adding 3 and 5mM ACD, whereas that for blood was significantly increased with obstruction of microchannel and adhesion of WBCs on the microchannel terrace after adding ACD. Our study suggested that 3 and 5mM ACD might not affect the passage time of RBC suspension and RBC deformability measured by MC-FAN.

L-cysteine improved ACD-induced blood fluidity impairment. L-cysteine significantly decreased blood passage time with elimination of ACD-induced microchannel obstruction and WBC adhesion on the microchannel terrace. However, no significant effects were observed in the passage time for RBC suspension after adding ACD and L-cysteine. The mechanisms by which L-cysteine improved blood fluidity impaired by ACD are unknown, but L-cysteine may act directly on ACD. Tsukamoto et al. reported that pre-administration of L-cysteine decreased serum ACD levels in ethanol-administered mice [17]. Moreover, L-cysteine was able to bind covalently with ACD, thereby forming a stable compound, nontoxic 2-methyl-thiazolidine-4-carboxylic acid [18,19].

We examined the *in vitro* effects of ACD and L-cysteine on blood fluidity. Therefore, further study will be needed to clarify whether alcohol consumption increases blood passage time. In addition, the effect of oral intake of L-cysteine on alcohol consumption-induced increase in blood passage time should be evaluated in detail. It was reported that blood passage time was a predictor of primary cardiovascular events in patients with traditional cardiovascular risk factors [8]. Our study might provide the basis for the conduction of a new clinical study to determine whether L-cysteine can prevent cardiovascular events caused by alcohol consumption.

This study suggested that blood fluidity, impaired by ACD, might improve after adding L-cysteine.

## Supporting information

S1 File Passage time for 100 μL blood following the addition of ACD and/or L-cysteine for all subjects. (XLSX)

S2 File Percentage of microchannels obstructed following the addition of ACD and/or L-cysteine for all subjects. (XLSX)

S3 File The number of adherent WBCs on the microchannel terrace following the addition of ACD and/or L-cysteine for all subjects. (XLSX)

S4 File Passage time for 100 μL RBC suspension following the addition of ACD and/or L-cysteine for all subjects. (XLSX)
